# Protein complexes identification based on go attributed network embedding

**DOI:** 10.1186/s12859-018-2555-x

**Published:** 2018-12-20

**Authors:** Bo Xu, Kun Li, Wei Zheng, Xiaoxia Liu, Yijia Zhang, Zhehuan Zhao, Zengyou He

**Affiliations:** 1 School of Software Technology, Dalian University of Technology, No.321 Tuqiang Road, Economic Development Zone, Dalian, 116024 China; 2 Key Laboratory for Ubiquitous Network and Service Software of Liaoning, Dalian, 116000 China; 3 College of Computer Science and Technology, Dalian University of Technology, No.2 Linggong Road, Ganjingzi District, Dalian, 116024 China; 4 College of software, Dalian JiaoTong University, Dalian, 116000 China

**Keywords:** Protein complexes identification, Protein-protein interaction network, Network embedding

## Abstract

**Background:**

Identifying protein complexes from protein-protein interaction (PPI) network is one of the most important tasks in proteomics. Existing computational methods try to incorporate a variety of biological evidences to enhance the quality of predicted complexes. However, it is still a challenge to integrate different types of biological information into the complexes discovery process under a unified framework. Recently, attributed network embedding methods have be proved to be remarkably effective in generating vector representations for nodes in the network. In the transformed vector space, both the topological proximity and node attributed affinity between different nodes are preserved. Therefore, such attributed network embedding methods provide us a unified framework to integrate various biological evidences into the protein complexes identification process.

**Results:**

In this article, we propose a new method called GANE to predict protein complexes based on Gene Ontology (GO) attributed network embedding. Firstly, it learns the vector representation for each protein from a GO attributed PPI network. Based on the pair-wise vector representation similarity, a weighted adjacency matrix is constructed. Secondly, it uses the clique mining method to generate candidate cores. Consequently, seed cores are obtained by ranking candidate cores based on their densities on the weighted adjacency matrix and removing redundant cores. For each seed core, its attachments are the proteins with correlation score that is larger than a given threshold. The combination of a seed core and its attachment proteins is reported as a predicted protein complex by the GANE algorithm. For performance evaluation, we compared GANE with six protein complex identification methods on five yeast PPI networks. Experimental results showes that GANE performs better than the competing algorithms in terms of different evaluation metrics.

**Conclusions:**

GANE provides a framework that integrate many valuable and different biological information into the task of protein complex identification. The protein vector representation learned from our attributed PPI network can also be used in other tasks, such as PPI prediction and disease gene prediction.

**Electronic supplementary material:**

The online version of this article (10.1186/s12859-018-2555-x) contains supplementary material, which is available to authorized users.

## Background

With the advent of the post-genomic era, the focus of life science research has shifted from genomics to proteomics. One important task in proteomics is to detect protein complexes from protein-protein interaction (PPI) networks. The discovery of protein complexes is not only critical to reveal the principle of cellular organization and functions, but also helpful to predict protein functions, disease genes and drug-disease associations. With the advances of high-throughput technologies, many large-scale PPI networks have been constructed [1, 2]. Hence, it is highly demanding to develop effective computational methods for the accurate identification of novel protein complexes.

In recent years, many computational methods have been proposed to predict protein complexes from PPI networks. A PPI network is usually modeled as an undirected graph, where the nodes in the graph represent proteins and the edges represent the interactions between proteins. Roughly, most of these protein complexes identification methods are based on the principle that densely linked regions in the PPI network correspond to actual protein complexes [3]. Therefore, the issue of predicting protein complexes can be formulated as the problem of detecting densely linked regions in PPI networks.

Existing computational methods for predicting protein complexes can be approximately divided into two broad categories [4]: (1) The methods based solely on PPI networks. These methods cluster the PPI network into multiple dense subnetworks only based on the topology of network [5]. They make use of merging, growing or partitioning strategies to detect protein complexes. Here, we just list a few typical methods in this category [6], e.g., CFinder [7], MCODE [8], LCMA [9], CMC [10], HACO [11], ClusterOne [12], MCL [13] and PEWCC [14]. (2) The methods based on PPI networks and some additional biological insights [15]. The biological insights are grouped as: core-attachment structure, evolutionary information, functional coherence, and mutually exclusive and co-operative interactions. CORE [16], COACH [17] and HUNTER [18] detected protein complexes based on the principle that each complex is composed by a core and its attachments. ProRank [19], ProRank+ [20] and the methods proposed by Sharan et al. [21, 22] detected conserved complexes across species based on the evolution of PPI networks. RNSC [23] and DECAFF [24] combined topological and GO information as functional information to detect complexes. Ozawa et al. [25] proposed a refinement method over MCODE and MCL to filter predicted complexes based on exclusive and co-operative interactions. Over the years, researchers tried to incorporate a variety of biological information to enhance the quality of predicted complexes [26]. However, it is still a challenge to integrate various biological evidences into a unified framework.

Recently, network embedding methods have shown to be effective in many graph data analysis tasks such as link prediction and network clustering [27]. Network embedding aims to represent nodes in the network in a low-dimensional space while preserving the node proximities. The definition of node proximities depends on the analytic tasks and application scenarios. According to the definition of node proximities, the state-of-the-art network embedding methods can be categorized into two groups: (1) structure-preserved network embedding; (2) attributed network embedding.

Structure-preserved network embedding methods focus on preserving the topological structure of the original network. Motivated by the similar power-law distribution of the vertices appearing in short random walk and the words in natural language, DeepWalk [28] regarded walks as the equivalent of sentences and then preserved the neighborhood structure of nodes by maximizing the co-occurrence probability between a target node and its context nodes within a truncated random walk window. Node2vec [29] proposed a method which can generate the neighborhoods of nodes using the 2nd order random walk. The LINE approach [30] solved the large-scale network embedding effectively by preserving the first and second order proximities.

Attributed network embedding targets at leveraging both the topological proximity and node/edge attribute affinity. MMDW [31] is a semi-supervised version of DeepWalk, which incorporates the labeling information into the network embedding. It jointly optimizes a maxmargin classifier and the representation learning model. By establishing the equivalent relationship between DeepWalk and matrix factorization, TADW [32] incorporates the rich text information into network embedding. AANE [33] is a scalable and efficient framework which learn a unified embedding representation by incorporating node attribute proximity into network embedding. It preserve the node proximity in both network structure space and attribute space.

The attributed network embedding method provides a general framework for incorporating both network structure information and additional node attribute information to generate a unified low-dimensional representation. This salient feature is particularly desirable in the context of protein complexes identification since using the additional biological information. The use of additional biological information sources often boost the identification performance significantly. Unfortunately, there are still no researches that exploit the attributed network embedding approach for protein complex detection. To fill this gap, we take the first attempt to investigate the feasibility and advantage of utilizing the attributed network embedding idea for protein complexes detection.

We propose a new method called GANE to predict protein complexes based on Gene Ontology(GO) attributed network embedding. The PPI network is represented as an attributed network in which the protein nodes are associated with GO slims. GANE first learns the vector representation for each protein from the GO attributed PPI network. Then, it uses a clique mining method to generate candidate cores. Consequently, a set of seed cores are generated from the set of candidate cores with density-based clique ranking and redundancy-based clique updating. For each seed core, its attachments are the proteins whose correlation score is larger than a threshold. The seed cliques with attachments are reported as the predicted protein complexes. In order to evaluate our method, we compared GANE with six classic protein complex identification methods, which are COACH [17], CMC [10], MCODE [8], ClusterOne [12], MCL [13] and PEWCC [14] on five different yeast PPI networks. Experiment results show that GANE performs better than the state-of-the-art methods with respect to different evaluation metrics. We summarize the contributions of this paper as follows:

- To our knowledge, although some methods incorporate several biological information in different ways, our method is the first piece of work that incorporates the attributed network embedding idea into the protein complexes identification problem.
- Our method provides a framework that integrate many valuable biological information into the task of protein complex identification.
- The protein vector representation learned from our attributed PPI network can also be used in other tasks, such as PPI prediction and disease gene prediction.

The remainder of the paper is organized as follows. In “Methods” section, we present the GANE method. We compare GANE with six classic complex identification methods and show the experiment results in “Results and discussion” section. Finally, “Conclusions” section gives a conclusion of this paper.

## Methods

**Fig. 1:**
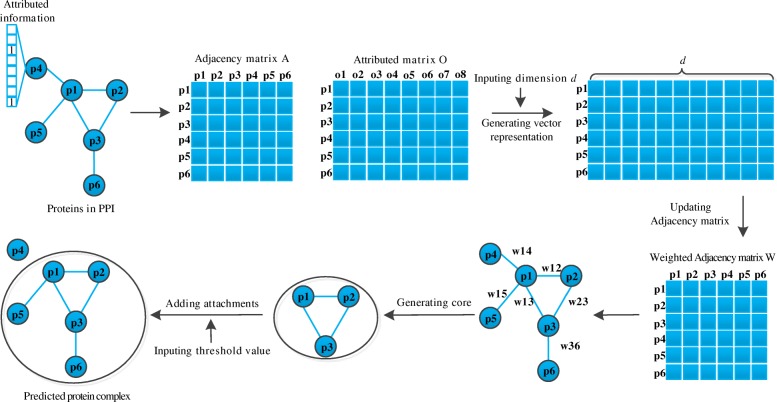
The basic idea of GANE to predict protein complexes from protein-protein interaction networks. The GANE method for protein complex prediction is a two-step procedure. Firstly, it learns the vector representation for each protein from the GO attributed PPI network. Based on the pair-wise vector representation similarity, a weighted adjacency matrix is constructed. Secondly, it uses a clique mining method to generate candidate cores. A set of seed cores are generated from the set of candidate cores with density-based clique ranking and redundancy-based clique updating. For each seed core, its attachments are those proteins with correlation scores that are larger than a threshold. The seed cores with attachments are the predicted protein complexes

The GANE method for protein complex prediction is a two-step procedure. Firstly, it learns the vector representation for each protein from the GO attributed PPI network. Based on the pair-wise vector representation similarity, a weighted adjacency matrix is constructed. Secondly, it uses a clique mining method to generate candidate cores. A set of seed cores are generated from the set of candidate cores with density-based clique ranking and redundancy-based clique updating. For each seed core, its attachments are the proteins whose correlation score is larger than a threshold. The seed cores with attachments are the predicted protein complexes. Figure 1 illustrates the basic pipeline of the GANE method in a vivid manner. Meanwhile, the major steps of our algorithm are presented in Table 1.

**Table 1:** Major steps of GANE

Algorithm 1 Protein complex identification algorithm GANE
Input: Graph *G* = (*V*, *E*), GO property matrix *O*, vector representation dimension *d*, threshold value *θ*
Output: A set of discovered protein complexes
Description:
Constructing a protein attribute affinity matrix *S*∈*R*^*n*×*n*^
Generating vector representation for each protein *φ*∈*R*^*d*^
Constructing a weighted adjacency matrix *W*
Initializing *Alternative_core*, *Seed_core*, *ComplexSet* to be $ \varnothing $
Generating maximal cliques and put them into *Alternative_core*
While $\textit {Alternative\_core}\ne \varnothing $:
DescendSort(*Alternative_core*) by *density_score*
*Alternative*_*core*=*Alternative*_*core*−*Clique*_1_
*Seed*_*core*=*Seed*_*core*+*Clique*_1_
Pruning and updating remaining cliques in *Alternative_core*
End while
For core *core*_*i*_ in *Seed_core*
finding the set of its attachments *Att*_*i*_
*ComplexSet*=*ComplexSet*+ *core*_*i*_∪*Att*_*i*_
End for
Return *ComplexSet*

### Learning vector representations for proteins

The network embedding technique transforms graph-structured data into vectorial data by learning the low-dimensional vector representation for each node in the network. Among existing network embedding methods, the AANE [33] aims at preserving both the topological similarity and node attribute similarity in the transformed space. Based on the AANE, we learn vector representations for proteins in the PPI network by preserving the proximities among proteins with respect to both the topological structure and GO attributes. The corresponding representation learning method is described below.

#### Topological model

In the GANE, a PPI network is represented as an undirected graph *G* = (*V*, *E*), where the nodes in *V* represent proteins and the edges in *E* represent the interactions between proteins. In order to preserve the topological proximity between proteins in the original PPI network, a loss function is defined as:

1 $$ \ell_{1} =\sum_{i \in V}\sum_{j \in V}a_{ij}\left(\varphi_{i}-\varphi_{j}\right)^{2},  $$

where *φ*_*i*_ and *φ*_*j*_ are the vector representations of protein *i* and protein *j*, the matrix *A*∈*R*^*n*×*n*^ represents the adjacency matrix of the PPI network, *a*_*ij*_=1 only if there is an interaction between protein *i* and protein *j*. Minimizing the penalty part *a*_*ij*_(*φ*_*j*_−*φ*_*j*_)^2^ means to minimize the embedding difference between *φ*_*i*_ and *φ*_*j*_ when *a*_*ij*_=1. Hence, proteins with similar topological structures will be forced to have similar vector representations. As a result, this model preserve the topological structures of the original PPI network.

#### GO attributed model

Gene Ontology (GO) is currently one of the most comprehensive ontology databases in the bioinformatics community [34]. It provides GO terms to describe three different aspects of gene product features: biological process (*Bp*), molecular function (*Mf*), and cellular component (*Cc*). GO slims is the cut-down version of GO, it contains a subset of the terms in the whole GO. They provide an overview on the ontology content without the details of the specific fine grained terms. GO slims give a comprehensive description on the biological attributes of proteins. Since GO slims of *Cc* include some protein complexes information, we only select GO slims of *Bp* and *Mf* as protein attributes.

After getting the attributed information for all the proteins in the PPI network, we generate an attribute matrix *O*∈*R*^*n*×*m*^, where *n* represents the number of proteins and *m* represents the number of GO slims attributes. Each entry *o*_*ij*_ in the matrix *O* describes whether protein *i* has a corresponding GO slim *j* or not with *o*_*ij*_=1 or 0. Based on the matrix *O*, we construct a protein attribute affinity matrix *S*∈*R*^*n*×*n*^. Each entry *s*_*ij*_ is calculated as below:

2 $$ s_{ij} =\frac{{\sum\nolimits}_{k=1}^{m} o_{ik}\times o_{jk}}{\sqrt{{\sum\nolimits}_{k=1}^{m} o_{ik}^{2}} \times \sqrt{{\sum\nolimits}_{k=1}^{m} o_{jk}^{2}}}.  $$

To preserve the proximity with respect to protein attributes, a loss function is defined as:

3 $$ \ell_{2} =\sum_{i \in V}\sum_{j \in V}\left(s_{ij}-\varphi_{i}\varphi_{j}^{T}\right)^{2},  $$

where *S*∈*R*^*n*×*n*^ is the protein attribute affinity matrix. Minimize this loss function means minimize the difference between the dot product of the vector representation *φ*_*i*_ and *φ*_*j*_ with the corresponding attribute similarity *s*_*ij*_.

#### Joint model for representation learning

Since topological and biological properties are both important for protein complexes identification, we use the topological model and GO attributed model together to learn the representations of proteins. The final loss function is defined as:

4 $$ \ell =\sum_{i \in V}\sum_{j \in V}a_{ij}\left(\varphi_{i}-\varphi_{j}\right)^{2} + \lambda \sum_{i \in V}\sum_{j \in V}\left(s_{ij}-\varphi_{i}\varphi_{j}^{T}\right)^{2},  $$

where *λ* is a parameter that controls the trade-off between topological and GO attributed properties. Since *ℓ* is separable for *φ*_*i*_, the corresponding minimization problem can be reformulated as a bi-convex optimization problem. T‘he original embedding problem is split into *2n* small convex optimization sub-problems. As shown in AANE [33], the distributed convex optimization technique ADMM [35, 36] can be used to solve this optimization problem. In each iteration, the *n* updating steps of *φ*_*i*_ is assigned to different workers in a distributed way. The distributed algorithm is guaranteed to converge to a local optimal point [35]. After solving the optimization problem of minimizing the loss function in Eq. (4), each protein is represented as a vector *φ*∈*R*^*d*^, where *d* represents the length of the embedding representation.

#### Weighted adjacency matrix

After obtaining the vector representation of each protein *φ*∈*R*^*d*^, we generate a weighted adjacency matrix *W*∈*R*^*n*×*n*^ as below, where *cos_sim* is the function for calculating the cosine similarity between two connected proteins based on the embedding representations.

5 $$ w_{ij} =\left\{ \begin{array}{lr} cos\_sim\left(\varphi_{i},\varphi_{j}\right) & a_{ij}=1 \\ 0 & a_{ij}=0 \end{array}. \right.  $$

### Clustering based on core-attachment structure

Gavin et al. [37] proposed that a protein complex is usually composed of two parts, a core and its attachments. Based on this principle, we detect protein complexes in two phases. Firstly, a set of seed cores are generated. Secondly, the attachments are included into each core based on their correlation strengths.

#### Generating cores

To generate cores, we use the cliques mining algorithm proposed by Tomita et al. [38] to enumerate all maximal cliques with at least three nodes in a PPI network. These cliques are considered as the candidate cores and we collected them into a *Alternative_core* set. Since not all the cliques in *Alternative_core* are suitable to be the cores of protein complexes, we prune the *Alternative_core* set to generate the *Seed_core* set based on the following procedure:

1. Cliques in *Alternative_core* are sorted in the descending order by *density_score*, denoted as *Clique*_1_,*Clique*_2_,…,*Clique*_*c*_. This *density_score* function considers both the inside connective density and biological correlation of each clique.
  6 $$ \textit{density\_score}(Clique_{q}) =\sum_{i,j \in Clique_{q}} w_{ij}.  $$
2. Remove *Clique*_1_ from *Alternative_core* and put it into the *Seed_core* set.
3. For any other clique *Clique*_*i*_∈*Alternative*_*core* that has an overlap with *Clique*_1_, *Clique*_*i*_ is updated with *Clique*_*i*_−*Clique*_1_. After that, if |*Clique*_*i*_|<3, remove *Clique*_*i*_ from *Alternative_core*.

This process repeats until *Alternative_core* is empty. The cliques in *Seed_core* are regarded as the core proteins in protein complexes.

#### Adding attachments

To detect attachments for each core, we focus on the strength of topological and biological connectivity between the core and the corresponding attachments. The correlation score between a clique in the *Seed_core* and a candidate attachment protein is calculated as below:

7 $$  {correlation\_score}\left(p_{i},Clique_{j}\right) = \frac{\sum_{k \in Clique_{j}} w_{ik}}{\left| Clique_{j} \right|},  $$

where protein *p*_*i*_ is one of the neighbors of the corresponding core *Clique*_*j*_. If the correlation score between protein *p*_*i*_ and *Clique*_*j*_ is larger than a threshold value *θ*, *p*_*i*_ is considered as one attachment of the corresponding clique.

Finally, each protein complex is generated by combining the core and its corresponding attachments.

## Results and discussion

### Datasets

Five yeast PPI networks were used in the performance comparison: DIP [39], Krogan-core [40], Krogan14k [40], Biogrid [41], Collins [42]. The detailed information of these five datasets are shown in Table 2. The GO slim information was downloaded from the website https://downloads.yeastgenome.org/curation/literature/go_slim_mapping.tab. To compare the predicted results with the reference complexes, we have constructed a standard complexes set by selecting all the protein complexes that had at least three proteins from MIPS, CYC2008, SGD, Aloy and TAP06. Consequently, there was a total 789 protein complexes in the reference set.

**Table 2:** The PPI data sets used in the experiment

PPI networks	Number of proteins	Number of interactions	Average clustering coefficient	Average number of neighbors
DIP	4928	17,201	0.095	6.981
Krogan-core	2708	7123	0.188	5.261
Krogan14k	3581	14,076	0.122	7.861
Biogrid	5640	59,748	0.246	21.187
Collins	1622	9074	0.555	11.189

Five yeast PPI networks were used in the performance comparison: DIP (Xenarios et al., 2002), Krogan-core (Krogan et al., 2006), Krogan14k (Krogan et al., 2006), Biogrid (Stark et al., 2006), Collins (Collins et al., 2007)

### Evaluation metrics

To formally evaluate the performance of our method, we use the same evaluation metrics as other methods [12, 14].

Let *P* denotes the set of predicted protein complexes from one method, the performance of this methods is mainly determined by the number of matched complexes between *P* and the set of gold standard protein complexes *B*. To determine if a predicted protein complex *p*∈*P* matches a known protein complex *b*∈*B*, we use the neighborhood affinity score *NA(p,b)* defined in in Eq. (8):

8 $$  NA(p,b) = \frac{{\left| V_{p}\cap V_{b} \right|}^{2}}{\left| V_{p} \right| \times \left| V_{b} \right|},  $$

where *V*_*p*_ is the set of proteins in the predicted protein complex *p* and *V*_*b*_ is the set of proteins in the reference protein complex *b*. Following the previous studies, *p* and *b* are considered to matched if *NA(p,b)* is larger than 0.25.

Based on the neighborhood affinity score, *N*_*cp*_ is defined as the number of predicted complexes that match at least one real complex, and *N*_*cb*_ is the number of real complexes that match at least one predicted complex.

9 $$  N_{cp} = \left| \left\{ p|p\in P,\exists b\in B,NA(p,b)\ge \omega \right\} \right|,  $$

10 $$  N_{cb} = \left| \left\{ b|b\in B,\exists p\in P,NA(p,b)\ge \omega \right\} \right|.  $$

In Eqs. (9) and (10), *ω* is threshold parameter, which is typically specified to be 0.25.

The first three measures used in the experiments for evaluating the performance of different methods are *Precision*, *Recall* and *F-score*. *Precision* is the proportion of predicted protein complexes that match at least one reference complex. *Recall* is the proportion of reference protein complexes that match at least one predicted complex. *F-score* is the harmonic mean of *Precision* and *Recall*.

11 $$  {Precision} = \frac{N_{cp}}{\left| P\right|},Recall = \frac{N_{cb}}{\left| B\right|},  $$

12 $$  {F-score} = \frac{2 \times {Precision} \times {Recall}}{{Precision}+{Recall}}.  $$

The other three metrics we used are clustering-wise sensitivity (*Sn*), clustering-wise positive predictive value (*PPV*) and geometric accuracy (*Acc*). Given |*B*| reference complexes and |*P*| predicted complexes, let *T*_*ij*_ denote the number of proteins that are found both in reference complex *i* and predicted complex *j*, and let *N*_*i*_ denote the number of proteins in reference complex *i*. Then, *Sn*, *PPV*, *Acc* are defined as follows:

13 $$  {Sn} = \frac{{\sum\nolimits}_{i=1}^{|B|} \max_{j=1}^{|P|} \left\{ T_{ij} \right\}}{{\sum\nolimits}_{i=1}^{|B|} N_{i}},  $$

14 $$  {PPV} = \frac{{\sum\nolimits}_{j=1}^{|P|} \max_{i=1}^{|B|} \left\{ T_{ij} \right\}}{{\sum\nolimits}_{j=1}^{|P|} {\sum\nolimits}_{i=1}^{|B|} T_{ij}},  $$

15 $$  {Acc} = \sqrt{{Sn} \cdot {PPV}}.  $$

### Performance comparison

**Fig. 2:**
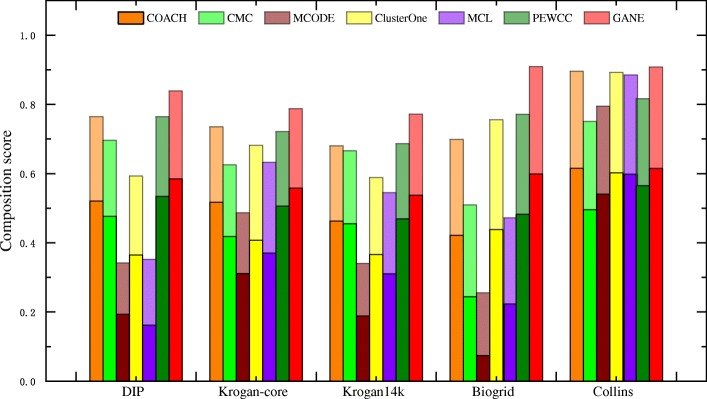
Comparison with six protein complex detection algorithms in terms of the composite score of F-score and Acc. Shades of the same color indicate different evaluating scores. Each bar height reflects the value of the composite score

For evaluating the performance of our algorithm, we compared our algorithm with six state-of-the-art protein complexes detection methods: COACH, CMC, MCODE, ClusterOne, MCL and PEWCC. The parameters of these methods were set as the default values as mentioned in their original papers. The embedding dimension *d*, the harmonic value *λ* and the threshold value *θ* of GANE were set to be 128, 0.1 and 0.3 respectively. For a fair comparison, we filtered out the complexes whose sizes are less than 3 in all algorithms. All experimental results were listed in Table 3 and Fig. 2.

**Table 3:** Performance comparison based on six evaluation metrics on the five yeast data

Datasets	Methods	#predicted complexes	#matched complexes	*Precision*	*Recall*	*F-score*	*Acc*
DIP	COACH	570	263	0.450	0.620	0.521	0.243
	CMC	179	108	0.603	0.394	0.477	0.219
	MCODE	59	32	0.542	0.118	0.194	0.149
	ClusterOne	341	133	0.390	0.343	0.365	0.227
	MCL	451	69	0.153	0.172	0.162	0.190
	PEWCC	666	413	0.620	0.469	0.534	0.230
	GANE	324	202	0.623	0.550	**0.584**	**0.254**
Krogan-core	COACH	348	206	0.592	0.460	0.518	0.217
	CMC	128	86	0.672	0.304	0.419	0.206
	MCODE	71	52	0.732	0.198	0.311	0.176
	ClusterOne	522	190	0.364	0.464	0.408	**0.273**
	MCL	376	126	0.335	0.414	0.371	0.262
	PEWCC	630	425	0.675	0.406	0.507	0.214
	GANE	208	161	0.774	0.436	**0.558**	0.229
Krogan14k	COACH	570	263	0.461	0.465	0.463	0.217
	CMC	396	187	0.472	0.440	0.455	0.210
	MCODE	49	30	0.612	0.112	0.189	0.152
	ClusterOne	225	105	0.467	0.302	0.366	0.222
	MCL	445	133	0.299	0.323	0.311	0.233
	PEWCC	934	500	0.535	0.418	0.470	0.217
	GANE	247	169	0.684	0.442	**0.537**	**0.234**
Biogrid	COACH	1507	469	0.311	0.657	0.422	0.276
	CMC	1503	236	0.157	0.553	0.245	0.265
	MCODE	58	16	0.276	0.043	0.075	0.181
	ClusterOne	476	187	0.393	0.497	0.439	**0.316**
	MCL	338	77	0.228	0.219	0.223	0.249
	PEWCC	2781	1044	0.375	0.677	0.483	0.288
	GANE	637	347	0.545	0.664	**0.599**	0.310
Collins	COACH	251	188	0.749	0.522	0.615	0.280
	CMC	153	104	0.680	0.390	0.496	0.255
	MCODE	111	94	0.847	0.400	0.540	0.254
	ClusterOne	195	143	0.733	0.511	0.602	0.290
	MCL	183	134	0.732	0.506	0.598	0.286
	PEWCC	570	477	0.837	0.426	0.564	0.252
	GANE	199	163	0.819	0.491	**0.615**	**0.293**

Both *F-score* and *Acc* are overall evaluation metrics, so the highest values of *F-score* and *Acc* are set in bold for each dataset

As shown in Table 3, GANE achieved the highest *Precision* and *F-score* on four data sets: DIP, Krogan-core, Krogan14k and Biogrid. It did not achieve the best *Precision* on the Collins dataset, but had the highest *F-score*. GANE did not achieve the highest *Recall*, probably because its number of predicted protein complexes is small. Overall, GANE performed the best with respect to the overall evaluation metric *F-score* for all datasets. In addition, our method reported the highest *Acc* value on all datasets except for Krogan-core and Biogrid. For these two datasets, ClusterOne was the best with respect to *Acc*. The ClusterOne method detected protein complexes based on seeding and greedy growth. So, the protein complexes detected by ClusterOne generally had more proteins, and its *Acc* was higher than that of our method. But the *Precision* and *F-score* of Clusterone were all lower than our method.

In order to visually observe the comparative results, Fig. 2 showed the composition score (*F-score* + *Acc*) of each method. In Fig. 2, the y-axis represented the sum of *F-score* and *Acc*. As shown in Fig. 2, our method always obtained the highest composition score. Therefore, our method outperformed other algorithms for all five datasets.

To examine the biological sense of the predicted protein complexes generated by GANE, we calculated the *P*-value by the tool GO::TermFinder [43]. Some of our unmatched predicted complexes actually had high biological significance. Due to the gold-standard complex set was still incomplete, these unmatched predicted complexes might be the new complexes that had not been discovered. Table 4 presented some case studies of GO analysis results from the DIP network. The min *P*-value represented the minimum *P*-value of the matched GO analysis results, it indicated that the collective occurrence of these proteins in a complex did not occur merely by chance. Thus, the predicted complex had a high probability to be real.

**Table 4:** Examples of predicted complexes on the DIP dataset

ID	Protein complex	Matched or not	Min *P*-value	GO-Description
1	YLR376C YHL006C YIL132C YDR078C	No	1.95e-10	DNA recombinase assembly
2	YFR015C YJL137C YLR258W	No	9.79e-07	Glycogen biosynthetic process
3	YLR078C YLR026C YDR189W YDR498C YLR268W YOR075W	No	1.42e-12	Vesicle fusion
4	YDR331W YMR298W YKL008C YHL003C YGR060W	No	3.96e-07	Ceramide biosynthetic process
5	YLR409C YER082C YKR060W YJR002W YPR144C YER127W YNL132W YDR299W YNL308C YCL059C YJL069C YCR057C YDR324C YGR145W	No	6.24e-23	Ribosomal small subunit biogenesis
6	YOR016C YHR140W YHL042W YBR106W YCR101C YDR414C YEL017C-A YAR028W YGL259W YKL065C YGL042C YER039C YJL004C YPL264C	No	0.00014	Protein localization to endoplasmic reticulum

Expect GO slims, we also utilized gene expression profile as attribute information for complexes detection. The performances were shown in Additional file 1: Table S1.

### Parameter sensitivity

In this part, we examined the sensitivity of GANE with respect to three parameters: the length of vector representation *d*, the harmonic value *λ* and the threshold value *θ*.

#### Effect of the embedding vector dimension

**Fig. 3:**
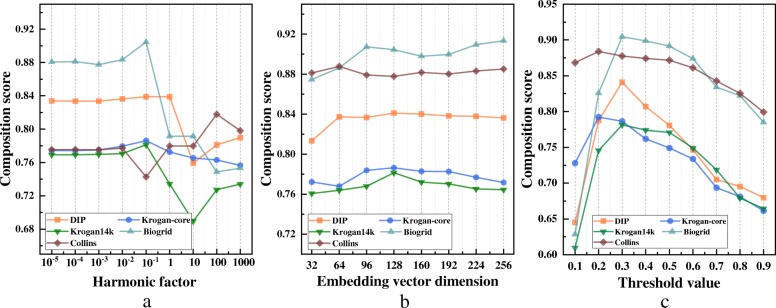
The sensitivity of GANE with respect to three parameters. **a** The performance of GANE when embedding vector dimension *d* was varied from 32 to 224. **b** The performance of GANE when harmonic value *λ* was varied from 0.00001 to 1000. **c** The performance of GANE when threshold value *θ* was varied from 0.1 to 0.9

In the experiment, the embedding vector dimension *d* was varied from 32 to 224. Figure 3a showed that the performance of our method was not very sensitive to the dimension parameter. Although the best results on different datasets were achieved with different dimension parameters, 128 was relatively a good choice in practice.

#### Effect of the harmonic value

The harmonic factor *λ* balanced the contributions of topological and biological information for GANE. To investigate the impact of *λ*, we varied it from 0.00001 to 1000. When *λ* was relatively low, topological information contributed much to the performance of our method. With the increasing of *λ*, biological information contributed much. As shown in Fig. 3b, different datasets achieved optimal solution with different *λ*. Here, we set *λ*=0.1 as default value.

#### Effect of the threshold value

The threshold value *θ* determined whether the neighbors of a core are included as its attachments. When the value *θ* was higher, it was harder for each neighbor to become an attachment. In other words, internal connections of the resulting protein complex were tighter. As shown in Fig. 3c, when *θ* was less than 0.1, the performance was relatively low. This was because when *θ* was small, most of the neighbors can be regarded as the corresponding attachments. The performance reached its peak when *θ*=0.3, so we set 0.3 as its default value.

## Conclusions

In this article, we propose an efficient method called GANE to detect protein complexes from PPI networks. GANE integrates biological evidences into the detecting process by learning vector representations for proteins from GO attributed network. As experimental results shown, GANE outperforms six protein complex detection methods on five different datasets. We concluded that the GO attributed network embedding can effectively enhance the quality of predicted complexes.

In the future, we will focus on investigating the following two questions:

1. How to learn the vector representations by incorporating more biological attributes in the PPI network? The incorporation of more biological evidences will further boost the identification performance.
2. How to apply the attributed network embedding methods to other biological networks, such as drug-drug interaction network and gene-phenotype network

## Additional file

Additional file 1 **Table S1.** The performances of GANE with different attribute information. (DOCX 25 kb)

## Abbreviations

Acc: Geometric accuracy
Bp: Biological process
Cc: Cellular component
GO: Gene Ontology
Mf: Molecular function
PPI: Protein-protein interaction
PPV: Clustering-wise positive predictive value
Sn: Clustering-wise sensitivity
