# Supplementary material for: Protein complexes identification based on go attributed network embedding
Source: BMC Bioinformatics. 2018 Dec 20;19:535. doi: 10.1186/s12859-018-2555-x (PMC6302388; doi:10.1186/s12859-018-2555-x)
Supplement: Supplementary file 1 — Table S1. The performances of GANE with different attribute information. (DOCX 25 kb) [file 12859_2018_2555_MOESM1_ESM.docx]

Additional file 1

**Protein Complexes Identification Based on GO Attributed Network Embedding**

Bo Xu^1,2,*^, Kun Li^1^, Zheng Wei^3,4^, Xiaoxia Liu^3^, Yijia Zhang^3^, Zhehuan Zhao^1,2^, Zengyou He^1,2^

^1^ School of Software Technology, Dalian University of Technology, Dalian 116024, China

^2^ Key Laboratory for Ubiquitous Network and Service Software of Liaoning, Dalian 116000, China

^3^ College of Computer Science and Technology, Dalian University of Technology, Dalian 116024, China

^4^ College of software, Dalian JiaoTong University, Dalian 116000, China

Author e-mails:

Kun Li, [likun9432@sina.cn](mailto:likun9432@sina.cn)

Zheng Wei, <weizheng@mail.dlut.edu.cn>

Xiaoxia Liu, <liuxiaoxia@mail.dlut.edu.cn>

Yijia Zhang, <Zhyj@dlut.edu.cn>

Zhehuan Zhao, <z.zhao@dlut.edu.cn>

Zengyou He, <zyhe@dlut.edu.cn>

* Corresponding author:

Bo Xu, <boxu@dlut.edu.cn>

Table S1 The performances of GANE with different attribute information

| Network datasets | Attribute information | *Precision* | *Recall* | *F-score* | *Acc* |
| --- | --- | --- | --- | --- | --- |
| DIP | GO slims | **0.623** | **0.550** | **0.584** | **0.254** |
|  | Gene expression profile | 0.304 | 0.316 | 0.310 | 0.226 |
| Krogan-core | GO slims | **0.774** | **0.436** | **0.558** | **0.229** |
|  | Gene expression profile | 0.627 | 0.349 | 0.448 | 0.214 |
| Krogan14k | GO slims | **0.684** | **0.442** | **0.537** | **0.234** |
|  | Gene expression profile | 0.397 | 0.289 | 0.334 | 0.199 |
| Biogrid | GO slims | **0.545** | **0.664** | **0.599** | **0.310** |
|  | Gene expression profile | 0.276 | 0.379 | 0.319 | 0.256 |
| Collins | GO slims | **0.819** | **0.492** | **0.615** | **0.293** |
|  | Gene expression profile | 0.777 | 0.480 | 0.586 | 0.259 |
